# Association between miRNA signatures in serum samples from epidermal growth factor inhibitor treated patients and skin toxicity

**DOI:** 10.18632/oncotarget.27953

**Published:** 2021-05-11

**Authors:** Sarah Kemski, Vivien Molitor, Michael Steffens, Tim J. Nümm, Nadine Herrmann, Thorsten Hornung, Thomas Bieber, Christian Schumann, Volker Kächele, Thomas Seufferlein, Volker Heinemann, Catharina Scholl, Julia Carolin Stingl

**Affiliations:** ^1^Research Division, Federal Institute for Drugs and Medical Devices (BfArM), Bonn, Germany; ^2^Department of Dermatology and Allergy, Christine Kühne Center for Allergy Research and Education (CK-CARE), University Hospital-Bonn, Bonn, Germany; ^3^Department of Pulmonology, Thorax Oncology, Sleep and Respiration Medicine, Hospital Group Allgäu, Kempten, Germany; ^4^Medical Centre for Haematology and Oncology, Ulm, Germany; ^5^Department of Internal Medicine I, University of Ulm, Ulm, Germany; ^6^Department of Internal Medicine III, Ludwig-Maximilians-University of Munich, Munich, Germany; ^7^Institute of Clinical Pharmacology, University Hospital of the RWTH Aachen, Aachen, Germany

**Keywords:** miRNA, EGFR, EGFRI, cancer, skin toxicity

## Abstract

Objective: Epidermal growth factor receptor inhibitors (EGFRI) are used as targeted cancer therapy. On average 70% of patients treated with EGFRIs suffer from skin toxicity. Studies showed a correlation between overall survival and the appearance of a skin rash, which is used as a biomarker for therapy efficacy. Micro RNAs (miRNA) as tumor or resistance biomarkers for cancer therapy are also highly investigated. In our study, we searched for associations of miRNA expression profiles in serum, with the severity of skin rash, in order to identify tentative therapy predictive biomarkers.

Materials and Methods: Five candidate miRNAs were selected, based on an earlier *in vitro* next-generation-sequencing-experiment and after literature search. MiR-21, miR-31, miR-17, miR-106b and miR-520e were investigated in serum samples from patients (*n* = 254) treated with EGFRI. The quantitative expression of miRNA was tested for association with the occurrence/severity of the rash.

Results: In our cohort of patients treated with EGFR inhibiting monoclonal antibodies, miR-21 and miR-520e serum concentrations were negatively correlated with severity of skin rash (*p*-value 0.000582 and 1.53e-07 linear-trend-test) whereas for miR-31, a positive correlation was observed (*p*-value 9.01e-06 linear-trend-test).

Conclusions: This suggests that miR-21, miR-31 and miR-520e expression might be a treatment dependent marker for EGFRI induced skin rash.

## INTRODUCTION

### EGFR-Inhibitors

Epidermal growth factor receptor inhibitors (EGFRI) belong to the so called targeted cancer therapy and are used for the treatment of different cancer types like non-small-lung-cancer (NSLC), head-and-neck-cancer (head-and-neck-ca), colon-rectal-cancer (colon-ca) and pancreas-cancer (pancreatic -ca) [1–4]. In most of these cancer types the EGF receptor (EGFR) is over expressed but a basal physiological expression of the receptor can also be found in epithelial cells such as keratinocytes [5]. EGFRI can be divided into small molecule tyrosine kinase inhibitors (e.g., erlotinib and gefitinib), which bind to the intracellular tyrosine domain of the receptor and inhibit auto phosphorylation, and monoclonal antibodies (e.g., cetuximab and panitumumab), which block the ligand binding site of the receptor [1, 2, 6, 7]. About 70–80% of patients treated with an EGFRI develop a skin reaction within the second to fourth week of the treatment. The phenotype of EGFRI-induced skin reaction is characterized by inflammatory papulo-pustular follicular rash, skin xerosis, and pruritus occurring on the scalp, face, upper chest and back [8, 9]. Earlier studies showed a strong correlation between the appearance of the skin rash and improved tumor progression and overall survival (OS) of patients treated with EGFRI [10, 11]. Therefore, skin rash is currently used as a predictive biomarker for the efficacy of EGFRI therapy [11, 12]. However, severe skin reactions, especially grade 3 or 4, can cause a break or even the end of therapy. In order to reduce this burden different treatments to reduce skin rash are used in clinical practice. For example the Germane Cancer Association published a guideline for supportive care in 2016 including preemptive and reactive treatment. For a preemptive treatment, they recommend general behavior actions like sun protection and general skin care and an oral prophylaxis with tetracyclines. The recommended reactive treatment depends on the grade of skin rash. For grad 1 skin rash same measures as preemptive treatment plus topical antibiotics, for grade 2 same measures as grade 1 plus topic steroids; for grade 3 and 4 same measures as grad 2 plus systemic steroids or oral isotretionin (but not in combination with antibiotics) is recommended [13]. The treatment recommendations of the guideline are based on different evince levels. The highest evidence can be found for a preemptive treatment of EGFRI induced skin rash with oral tetracycline. For this treatment a double-blind placebo-controlled randomized trial and different randomized trials showed a significant reduction of the severity of skin toxicity in patients treated with tetracycline’s [14–17]. The treatment of already existing skin rash with topic corticosteroids, which have an anti-inflammatory and anti-pruritic effects, shows lower evidence [18]. Also with a low evidence, due to a missing control cohort in the study, orally administrated isotretinoin and clindamycin could reduce grade 2–3 skin rash to grade 0–1 [19]. But with all these possible treatments of EGFRI induced skin rash, its treatment predictive value might get lost and new biomarkers will be needed.

EGFR ligands like epiregulin (EREG), amphiregulin (AREG), and hepatocyte growth factor (HGF) were investigated as biomarkers for skin rash and found to be inversely proportional to grades of skin toxicity [20]. Also genetic variations were assessed as possible biomarkers but only a mutation in EGFR intron 1 showed significant associations toward skin toxicity [21].

The multi centric Dermatoxgen study was initiated by our research group with the aim to identify tentative biomarkers for prediction of the occurrence of EGFRI induced skin rash. Within the scope of this study, different aspects of the EGFRI-induced skin rash were investigated, all with the objective to better understand EGFRI induced skin rash and find predictive biomarkers. To avoid any bias in the appearance or grade of skin rash, patients in our study only received reactive treatment if necessary with topical corticosteroids, topical antibiotics, oral antibiotics and antihistamines. The study showed a correlation between the occurrence of the skin rash and the drug-metabolizing activity assessed by the erlotinib/O-desmethyl-erlotinib metabolic ratio [22]. An investigation of genetic variations showed that patients carrying the HLA-A^*^02:01 or HLA-A^*^03:01 alleles are less likely to develop a skin rash [23]. Also for the EGF receptor associations between skin toxicity of EGFRIs and mutations of the EGFR were found. For the SNP rs534124757 of the EGFR gene it was shown that in patients carrying at least one A allel skin toxicity was less frequently [24]. Deep sequencing of the EGFR gene locus and its downstream signaling pathway revealed also an association between a SNP rs2293348 in the EGFR gene and the rash [25]. But not only mutations in the EGFR itself seem to be associated with skin toxicity. A genotyping of cancer patients revealed an association between skin rash and a specific haplotype of phosphatidylinositol-3-kinase (PIK3CA) [24]. An investigation of biomarkers in the plasma of the Dermatogen study group showed that patients with a lower IL-8 concentration are in risk to develop a more severe skin rash but had a longer survival. The same was found for patients with a low HGF concentration in the plasma [26, 27].

### Gene regulatory biomarkers of EGFRI-induced skin rash

MicroRNAs (miRNAs) are short non-coding RNAs with a length of around 19–25 nucleotides, that are involved in the post transcriptional regulation of gene expression. One miRNA can regulate the expression of many different genes by inducing translational inhibition or transcript degradation. Therefore, miRNAs are important for cell processes like proliferation, differentiation, apoptosis, stress tolerance and immune response [28]. The role and machinery of miRNAs in skin cells have been broadly investigated [29]. MiR-21 for example is involved in migration processes of keratinocytes and fibroblasts [29]. Some miRNAs are up- or down-regulated in pathophysiological processes. In inflammatory skin diseases like psoriasis and atopic dermatitis, miRNAs are suggested to be involved in inflammatory processes and immune dysfunction [30, 31]. However, miRNAs are not only highly involved in skin physiology and in pathophysiology; they are also in the focus of research in the field of tumor diagnostic and prognostic biomarkers and the resistance to cancer therapy [32, 33]. A set of 5 miRNAs (mir-17, mir-660, mir-92a, mir-106a, and mir-19b) is most commonly dysregulated in serum and tumor tissue samples from lung cancer patients and has therefore promising diagnostic potential [34]. In a meta-analysis circulating miR-31 was determined as an effective biomarker for cancer detection and prognosis [35]. MiRNAs may also be markers for therapy resistance. Different miRNAs mediate drug resistance in colon cancer by distinct mechanisms [33]. 16 miRNAs were differentially expressed between EGFRI resistant patients and EGFRI sensitive patients and might therefor be dependent for the sensitivity to EGFRI treatment [36]. Cetuximab resistance of colon cancer patients is connected to an upregulation of miR-199a-5p and miR-375 targeting PH domain and leucine-rich repeat protein phosphatase 1 (PHLPP1) a tumor suppressor [37].

All this shows the involvement of miRNAs in physiological and pathophysiological processes in skin cells, especially in inflammatory diseases like psoriasis or atopic dermatitis, and their already frequently discussed role as biomarkers in cancer. This leads to the assumption that miRNAs might also be involved in the development of EGFRI-induced skin rash and therefor can be used as possible biomarkers for therapeutic resistance. However, so far most of the studies in cancer patients focused on miRNAs expressed in cancer cells or secreted into body fluids most likely by cancer cells, while miRNAs associated with the skin rash have not been investigated. To elucidate the influence of miRNAs in the development of skin rash we previously investigated the miRNA expression in fibroblasts incubated with and without erlotinib [38]. In this analysis now, we firstly identify the miRNA expression profiles in skin cells, namely keratinocytes and fibroblasts, depending on their capability to react to erlotinib incubation. From those we depicted a set of five miRNAs which concentrations were determined in serum samples, derived from EGFRI treated patients, and evaluated with respect to the appearance and severity of the skin rash.

## RESULTS

### Erlotinib induces a distinct miRNA profile in primary human keratinocytes and fibroblasts

**Figure 1:**
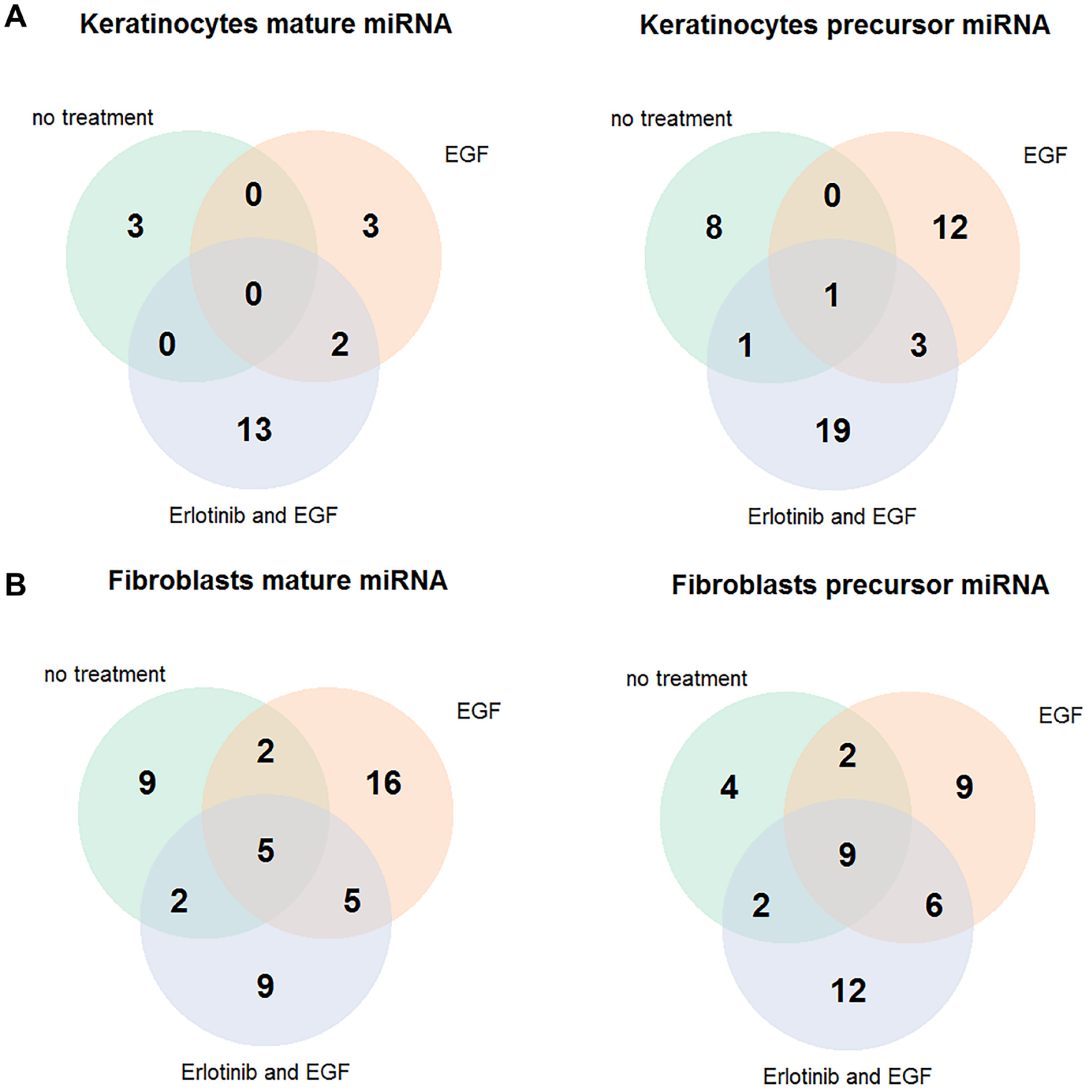
Erlotinib-sensitive keratinocytes and fibroblasts express a different miRNA pattern than those insensitive to erlotinib. Cells were treated with 5μM erlotinib or 0.05% DMSO and stimulated with 4 nM EGF. (**A**) Differentially expressed miRNAs between erlotinib sensitive and insensitive primary human keratinocyte cell samples (fold change ≥ 1.5 or fold change ≤ 0.66, *p*-value ≤ 0.05). (**B**) Differentially expressed miRNAs between erlotinib sensitive and insensitive human dermal fibroblast cell samples (fold change ≥ 1.5 or fold change ≤ 0.66, *p*-value < 0.01) Abbreviation: EGF: epidermal growth factor.

In order to gain a first impression about the effect of EGFRI incubation on the miRNA expression in skin cells, we studied effects of *in vitro* EGFR inhibition with the (small molecule) EGFRI erlotinib on miRNA expression in human keratinocyte/and fibroblast cell samples from healthy donors. Cells were classified into cell samples more reactive on erlotinib incubation and less reactive ones. Detailed information on the classification is provided in the supplements (Supplementary Figures 1 and 2). Fifty-four miRNAs were exclusively up or down regulated in the cell samples that were more reactive on erlotinib incubation compared to less reactive ones (fold change ≥ 1.5 and ≤ 0.66; 32 miRNAs in keratinocytes with *p*-value ≤ 0.05; 22 miRNAs in fibroblasts with *p*-value < 0.01) (Figure 1; Supplementary Tables 1 and 2). In order to decide, which of those 54 differentially expressed miRNAs were the most promising ones to be further investigated, a literature search on miRNAs involved in regulation of EGFR pathways was conducted. The search included all publications in which the miRNAs were connected to EGFR inhibitor treatment, erlotinib treatment in particular and cancer cells or bio fluid samples from cancer patients. According to our cell culture findings and the literature reports, five miRNAs (miR-31, miR-17, miR-106b, miR-520e, miR-21) were chosen as candidates to be further investigated in serum samples from EGFRI treated patients for potential correlations with the development of a skin rash.

### MiRNA serum levels of EGFRI treated patients are associated to EGFRI induced skin rash

Details about the patients who provided serum samples for the analysis of genetic and epigenetic biomarkers for EGFRI related skin rash from the Dermatoxgen study, are given in Tables 1 and 2. Patients were observed on week 1, 2, 3, 4, 26 and 53 after the start of the therapy. In week 4 serum, plasma and blood samples were taken. Serum samples were available from 254 patients and total RNA including miRNA was extracted.

**Table 1:** Patients characteristics (age, gender and smoking) and clinical characteristics (tumor type and skin rash)

Patient characteristics	Patients	TKI treated patients	EGFR-mAbs treated patients
**Sample size**	*N* (254)	*N* (156)	*N* (98)
**Age: Mean (SD)**	66.4 (9.8)	67.3 (9.0)	65.0 (10.8)
**Gender: *N* (%)**			
** Male**	161 (63.4)	92 (59.0)	69 (70.4)
** Female**	93 (36.6)	67 (41.0)	29 (29.6)
**Smoking: *N* (%)**			
** No**	94 (38.1)	52 (34.3)	42 (43.8)
** Yes (present)**	30 (12.1)	15 (9.9)	15 (15.6)
** Yes (former)**	123 (49.8)	84 (55.6)	39 (40.6)
** NA**	7	5	2
**Tumor Type:**			
** Lung-Cancer**	136 (53.5)	107 (68.6)	29 (29.6)
** Colon-Cancer**	58 (22.8)	2 (1.3)	56 (57.1)
** Head and Neck Cancer**	11 (4.3)	0	11 (11.2)
** Pancreatic Cancer**	49 (19.3)	47 (30.1)	2 (2.0)
**Skin Rash:**			
** No**	51 (20.1)	35 (22.4)	16 (16.3)
** Grade 1**	98 (38.6)	60 (38.5)	38 (38.8)
** Grade 2**	92 (36.2)	52 (33.3)	40 (40.8)
** Grade 3**	13 (5.1)	9 (5.8)	4 (4.1)
** Grade 4**	0	0	0

Abbreviations: EGFR-mAbs: epidermal growth factor receptor monoclonal antibodies; TKI: tyrosine kinase inhibitor; N: number of patients; NA: not kown.

**Table 2:** Distribution of therapeutic agents (EGFRIs) and cancer types in the patient cohort

	Cetuximab	Erlotinib	Gefitinib	Panitumumab	Sum
**Lung-Cancer**	29	94	13	0	136
**Colon-Cancer**	38	1	1	18	58
**Head and Neck Cancer**	11	0	0	0	11
**Pancreatic Cancer**	2	47	0	0	49
**Sum**	80	142	14	18	254

All five candidate miRNAs (miR-31, miR-17, miR-106b, miR-520e, miR-21), chosen from the *in vitro* NGS experiments and from the literature, were quantified in serum samples of EGFRI treated patients and correlated to the clinically observed severity and course of skin rash.

All four included EGFRIs have a skin rash as possible side effect and the skin rash appears tumor type and state independently. Therefor the association between the miRNA concentration and skin rash was analyzed independently from the tumor type and treatment, in all 254 patients.

Of the five miRNAs studied, miR-31, miR-21 and miR-520e showed correlations (*p*-value 0.00296, 0.0378 and 0.0199 [linear trend test], *p*-values not corrected for multiple testing) between serum concentration and the severity of the skin rash (Table 3).

**Table 3:** Associations between the serum concentration of the six tested miRNAs and the occurrence/severity of the skin rash

miRNA		All Patients (*N* 254)	EGFR-mAbs treated patients (*N* 98)	TKI treaded patients (*N* 156)
miR-17	**Appearance of skin rash**	*p* = 0.376	*p* = 0.264	*p* = .429
	**Severity of skin rash**	*p* = 0.594	*p* = 0.613	*p* = .62
miR-21	**Appearance of skin rash**	*p* = 0.098	***p* = 0.047 **	*p* = .439
	**Severity of skin rash**	***p* = 0.002 **	***p* < 0.001 **	*p* = .115
miR-31	**Appearance of skin rash**	*p* = 0.564	*p* = 0.081	*p* = .472
	**Severity of skin rash**	***p* = 0.037 **	***p* < 0.001 **	*p* = .43
miR-106b	**Appearance of skin rash**	*p* = 0.314	*p* = 0.938	*p* = .256
	**Severity of skin rash**	*p* = 0.182	*p* = 0.699	*p* = 0.168
miR-520e	**Appearance of skin rash**	*p* = 0.126	***p* = 0.044 **	*p* = 0.651
	**Severity of skin rash**	***p* = 0.019 **	***p* < 0.001 **	*p* = 0.473

Correlation between different miRNA serum levels in EGFRI treated patients and the occurrence or severity of the skin rash. Correlation was analyzed for the whole cohort and for subgroups divided between the treatments of the patients. Correlation was analyzed using linear-trend test. *P*-values were not corrected for multiple testing. Abbreviations: N: number of patients; EGFR-mAbs: epidermal growth factor receptor monoclonal antibodies; TKI: tyrosine kinase inhibitor.

MiR-21 and miR-520 were negatively correlated with skin rash and showed the lowest expression in patients with severe grade 3 skin rash.

MiR-31 was positively correlated with skin rash with the highest expression in patients with grade 3 skin rash. For miR-17 and miR-106b, no significant association with severity of skin rash was found (Table 3).

### Low miR-21, miR-520e and miR-31 serum concentrations are associated with longer survival

**Figure 2:**
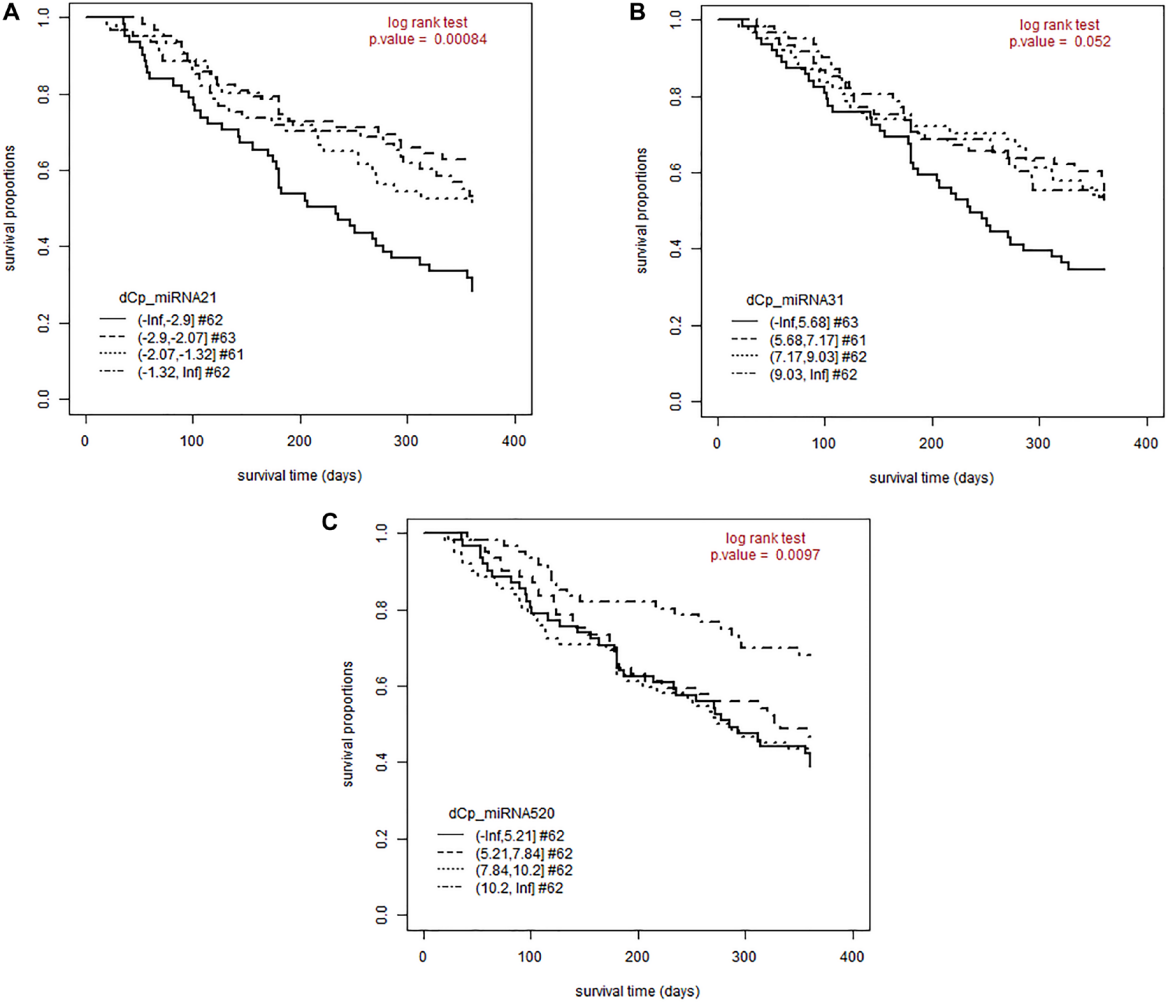
A lower miRNA concentration is associated with a longer survival for miR-21, miR-31 and miR-520e. Patients were followed-up for 360 days after initiation of EGFRI therapy. The proportion of patients still alive is plotted over the observation period. Patients were split by delta C*P* values of each miRNA into four equally-sized groups. (**A**) Kaplan-Meier- Plot for miR-21; log rank test, *p*-value < 0.001; mean OS for miR-21 concentration 1: 221 days (SE: 15.3); mean OS for miR-21 concentration 2: 290 days (SE: 13.3); mean OS for miR-21 concentration 3: 271 days (SE: 14.1); mean OS for miR-21 concentration 4: 275 days (SE: 15.0). (**B**) Kaplan-Meier- Plot for miR-31; log rank test, *p*-value = 0.055; mean OS for miR-31 concentration 1: 233 days (SE: 14.8); mean OS for miR-31 concentration 2: 272 days (SE: 14.9) ; mean OS for miR-31 concentration 3: 275 days (SE: 15.0); mean OS for miR-31 concentration 4: 275 days (SE: 14.0). (**C**) Kaplan-Meier- Plot for miR-21; log rank test, *p*-value = 0.0088; mean OS for miR-520e concentration 1: 247 days (SE: 15.0); mean OS for miR-520e concentration 2: 261 days (SE: 14.8); mean OS for miR-520e concentration 3: 245 days (SE: 15.7); mean OS for miR-520e concentration 4: 302 days (SE: 12.7) Abbreviations: ^#^: number of patients; dCP: delta crossing point; OS: overall survival.

For those three miRNAs, which were correlated to the severity of the skin rash, a survival curve analysis was performed. The Kaplan-Meier-plots showed a significant correlation between a low miRNA serum level and a longer overall survival for miR-21 and miR-520e (*p*-value 0.00046 and 0.0088) (Figure 2A and 2C). For miR-31 a trend could be seen between low miR-31 serum level and longer survival (*p*-value 0.055) (Figure 2B).

### Association between miR-21, miR-31 and miR-520e serum concentrations and skin rash is not treatment independent

**Figure 3:**
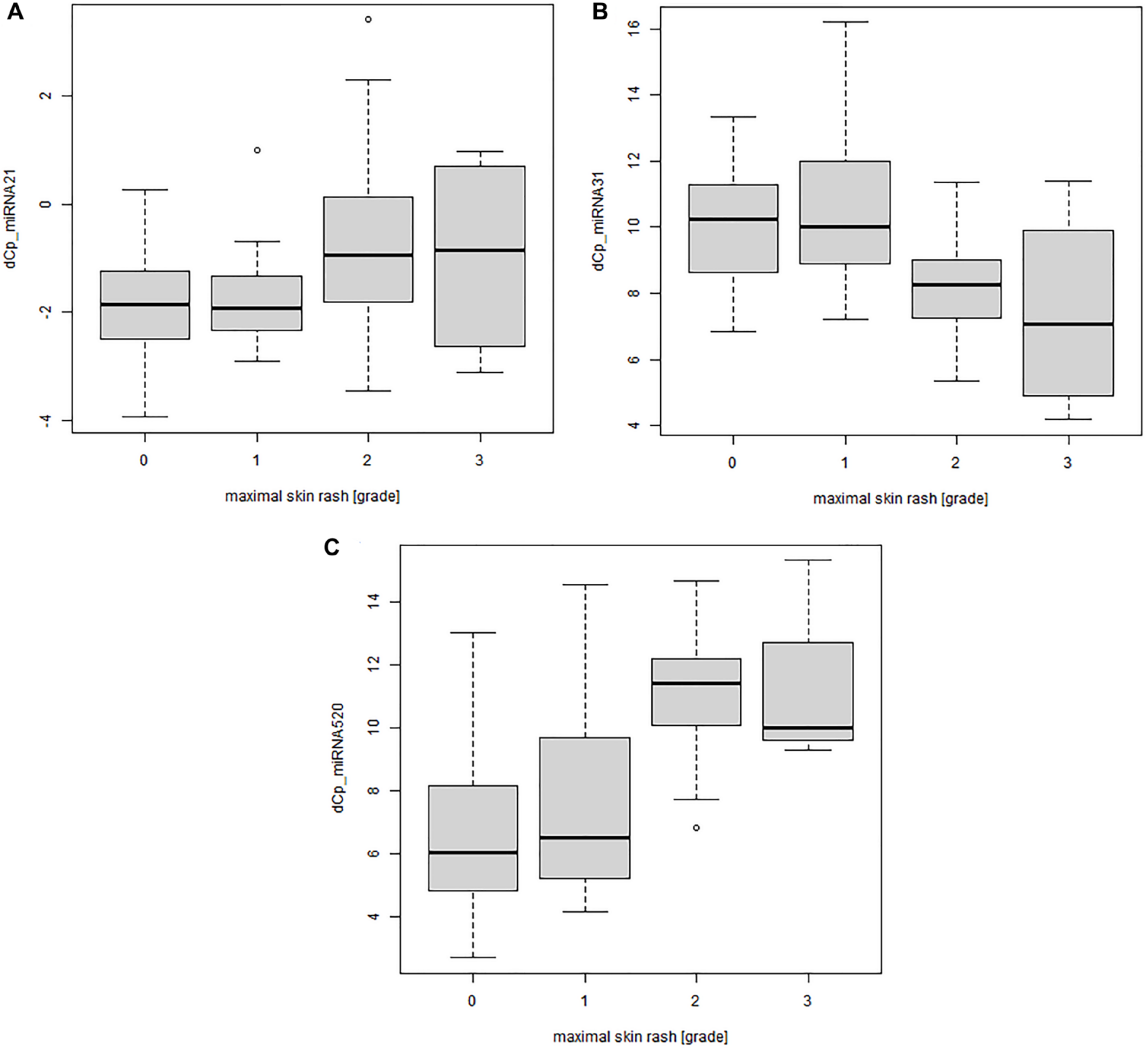
Significant correlation between miRNA serum concentration and severity of the skin rash for patients treated with monoclonal antibody EGFRIs. MiRNA concentrations were determined by qPCR (*n* = 98). dC*P* values were calculated against miR-93. A high dC*P* value means the miRNA is down regulated. (**A**) miR-21 concentration plotted against the maximum severity of the skin rash during observation period; linear trend test, *p*-value < 0.001. (**B**) miR-31 concentration plotted against the maximum severity of the skin rash during observation period; linear trend test, *p*-value < 0.001. (**C**) miR-520e concentration plotted against the maximum severity of the skin rash during observation period; linear trend test, *p*-value < 0.001. (0 = no skin rash, 1 = light skin rash, 2 = mild skin rash, 3 = sever skin rash). Abbreviation: dCP: delta crossing point.

Subgroup analyses of patients who had been treated with either small molecule or antibody tyrosine kinase inhibitors, revealed significant correlations between miR-21, miR-31, and miR-520e and the severity of the skin rash only in the antibody treatment group (*p*-values of 0.000582, 9.01e-06 and 1.53e-07 [linear trend test], not corrected for multiple testing) (Figure 3A–3C). As for the whole cohort a negative correlation of miR-21 and miR-520e serum concentration with the severity of the skin rash could be seen. For miR-31 a positive correlation with the severity of the skin rash was found. However, no significant correlation between miRNA serum concentration and skin rash in patients treated with small molecule tyrosine kinase inhibitors was observed.

## DISCUSSION

In our study, we analyzed the association between the typical skin rash developed under EGFRI therapy and the serum concentration of different miRNAs. Understanding this connection might lead to a better understanding of epigenetic mechanisms behind the skin rash and help to find predictive biomarkers for its development. Out of the five candidate miRNAs, chosen from a combination of literature search and prior *in vitro* experiments in a skin cell model, miR-21, miR-31 and miR-520e showed significant correlations between skin rash and serum concentration.

So far, there were many different studies searching for miRNAs as possible biomarkers in cancer development and disease outcome using tissue samples from the tumor, the surrounding tissue and/or blood samples [39, 40]. The objective of this study was to identify epigenetic miRNA associations with therapy-induced skin rash in patients receiving an EGFR targeting tumor treatment, since skin toxicity of EGFRI treatment has been shown to be a prognostic and therapy-predictive factor. In order to identify miRNAs that are involved in EGFR inhibition, we started by analyzing the effect of erlotinib incubation on the miRNA expression in primary human keratinocytes and fibroblasts from healthy donors. A literature search about which of the significantly regulated miRNAs from the *in vitro* experiments, were already connected to cancer and/or EGFRI therapy, gave us an idea, which miRNAs might be involved in the appearance of the skin rash and could potentially be found in serum samples from EGFRI treated patients.

Linear trend tests revealed negative correlations between the serum concentrations of miR-21 and miR-520e and the severity of the skin rash. Survival curve analysis also showed a negative correlation between miR-21 and miR-520e serum concentrations and overall survival time. For miR-31 a positive correlation between serum concentration and severity of the skin was observed. The survival curve for miR-31 showed a negative correlation between miR-31 serum concentration and survival time.

Because of the frequent mentioning of miR-21 in publications on miRNAs and cancer treatment resistance, this miRNA was the only one we investigated in our patient cohort even though it was not significantly differentially regulated in the *in vitro* cell experiments. Hua Shen et al. discovered that a high miR-21 and low phosphatase and tensin homolog (PTEN) concentration might indicate a poor gefitinib clinical response [41]. Another study investigated the miR-21 expression in colon-cancer tissue compared to normal adjacent tumor tissues. The colon-cancer tissue had higher miR-21 content and tumor patients with a lower miR-21 expression had a longer overall survival (OS) and disease-free survival (DFS) [41, 42]. These results in tumor tissue fit well to our finding that lower miR-21 concentration correlated with higher grade of skin rash, since skin rash has a positive prognostic value. In our cohort, we also observed a correlation between miR-21 in serum and a longer survival of patients. However, in our study we looked at treatment related outcome of the patients, and therefor included different tumor and disease stages. The Kaplan-Meier plot demonstrates that a low miR-21 serum concentration was correlated to a longer survival and miR-21 serum concentration was inversely correlated with the grade of skin rash. Our results suggest a better response to the EGFRI treatment in patients with a low miR-21 serum concentration.

MiR-520 is mostly studied in patients with non-small-lung-cancer. In NSLC metastasis miR-520 seems to be upregulated and by this down regulates transforming growth factor-β receptor 2 (TGFBR2) which regulates carcinogenesis and metastasis via the transforming growth factor-β (TGF-β) pathway [43]. In another study mmu-miR-291a-3p, a mouse homolog of miR-520e, inhibits cell senescence via the TGF-β receptor 2 signaling pathway by reducing protein expression of TGF-β receptor 2. Mmu-miR-291a-3p also seems to enhance the proliferative potential of senescent cells. [44]. In our results, patients with EGFR inhibitor induced skin rash had lower expression of miR-520e indicating a better prognosis, and this was especially significant in patients treated with monoclonal antibodies. In our study, we used serum samples instead of NSCLC tissue for miRNA isolation, but in principle, the better prognosis was correlated with lower serum concentrations, which fits well to the findings in lung tissue. In addition, the fact that the correlation between lower miR-520e serum concentrations and skin rash gets even more significant looking at patients treated with monoclonal antibodies, shows that low miR-520e in our study might be an indicator for good response to the therapy because the miR-520e we measured might not originate from tumor cells. We could also see in the Kaplan- Meier plots that patients with low miR-520e expression have a longer survival time. A possible link between the grade of skin rash and lower miR-520e expression might be TGF-β. Patients with atopic dermatitis seem to have a higher expression of TGF-β and other inflammatory cytokines like IL-6, IL-8 and IL-10 compared to healthy individuals [45].

For miR-31 a study by Ning Xu et al. showed an upregulation in keratinocytes from psoriasis patients. They demonstrated that TGF-β1, which is also upregulated in psoriasis epidermis, upregulates miR-31. Furthermore, an inhibition of miR-31 suppressed NF-kB–driven promoter luciferase activity and the basal and tumor necrosis factor-α (TNF-α) induced production of IL-1b, CXCL1/growth-related oncogene-a, CXCL5/epithelial-derived neutrophil-activating peptide 78, and CXCL8/IL-8 in human primary keratinocytes [46]. Regarding pathophysiology of the EGFRI induced skin rash, an inflammatory component is postulated by several publications. [8, 47, 48] This together with the effect of miR-31 on inflammatory cytokines is in line with our finding that a higher miR-31 concentration is associated with a higher grade of skin rash. A similar relationship can also be observed for miR-520e. On the other hand, we found a correlation between low miR-31 serum concentration and a longer survival. In a meta-analysis of 14 different studies including different tumor types, a significant correlation between low miR-31 concentration in blood and a longer overall survival was shown [35]. Studies in tumor biopsies confirm that a low miR-31 expression correlates with a longer overall survival [49]. Even for EGFR- inhibitor therapy a study comparing cetuximab treated patients with low or high miR-31 concentration in tumor tissue found that a low miR-31 expression leads to longer OS and PFS [50]. The differences in correlation between miR-31 serum concentration and skin rash or OS might lead to the assumption, that miR-31 could have a different effect on skin cells than tumor cells under EGFRI treatment. Both, the study by Anandappa et al. and the one by Laurent-Puig et al. used tumor tissue instead of serum samples. The meta-analysis by Ma et al. used blood samples but they did not take into account the treatment of the cancer patients. Those two differences might support the suggestion that the miR-31 expression we found is treatment dependent and that miR-31 has different effects on skin cells like keratinocytes or fibroblasts than on tumor cells. MiR-31 expression is elevated in keratinocytes incubated with inflammatory cytokines like TNF-α or interferon-γ [51]. Furthermore, an interferon-γ incubation of keratinocytes in which the EGFR was blocked resulted in an increased chemokine expression [52]. This might be an explanation for the correlation between high miR-31 serum concentration and high grade of skin rash reflecting the inflammatory component of the skin rash. Blocking the EGF receptor with monoclonal antibodies might lead to an inflammatory reaction in the skin cells, which in turn leads to an elevated miR-31 expression in skin cells, which then might release miR-31 to the blood.

Looking at our results a correlation between miRNA serum levels and the skin rash under EGFRI treatment can be found. For three out of five candidate miRNAs, a significant association between the miRNA concentration and the severity of the skin rash was shown especially for patients treated with monoclonal antibodies. This suggests a specificity of miR-21, miR-31 and miR-520e for the therapy with monoclonal antibodies and expression of the analyzed miRNAs most likely by cells other than tumor cells, such as skin cells. As a possible mechanism, an inflammatory component could be identified by looking at the miRNAs we analyzed and their connection to other skin diseases. Inflammation is already discussed to be part of the mechanism of EGFRI induced skin toxicity [48]. Taking all this together, it makes it quite interesting to further investigate the role of miR-21, miR-31 and miR-520e for the development of EGFR inhibitor induced skin rash in patients treated with monoclonal antibodies and to understand possible mechanisms behind it. A better understanding of the mechanism might than lead to possible predictive biomarkers for EGFRI induced skin rash, which are needed as an alternative to the skin rash is self as prognostic biomarker for therapy response. Because with a better treatment of the EGFRI induced skin rash it prognostic value might get lost.

### Limitations

The biggest limitation might be that there are no serum samples from patients before they were treated with an EGFR inhibitor. Hence, we cannot say if the effects we found are mostly due to the therapy with an EGFRI or if those effects can be seen in patients in general. However, because of the heterogeneity of the cohort concerning the tumor type and cancer state, a treatment specific effect is possible. Still these miRNAs give us a better understanding about the skin rash, which can be used for further investigations.

## MATERIALS AND METHODS

### *In vitro* miRNA profiling in human primary skin cells

#### Cell model

Primary human keratinocytes were isolated from skin samples derived from healthy controls of the Dermatox_Epigen study. This study aims to analyze epigenetic differences in skin samples of EGFR inhibitor treated cancer patients. As a control collective patients from the university women’s clinic Bonn were enrolled. These patients underwent plastic surgeries from which skin samples were derived to isolate keratinocytes. After obtaining the skin, a wash step was performed with a sterile gauze soaked in PBS to remove excess blood. Afterwards, the split thickness-skin (0.4 mm) was immediately prepared using a dermatome. The epidermal cell suspension was generated with freshly prepared trypsinization solution (PBS supplemented with 0.5% trypsin (Sigma-Aldrich) and 1% Antibiotic-Antimycotic (Gibco™, Waltham, MA, USA)). The floating split thickness-skin was incubated in the trypsin solution for 1 h at 37°C, whereby the dermal side was in contact with the trypsin solution. Afterwards, the dermis was removed and the epidermis was added to medium supplemented with 1% DNase to remove DNA and to avoid cell clumping. Next, the epidermal cell suspension was generated by several steps of pipetting up and down or by vortexing until the media became cloudy, indicating that the cells were in suspension. Written informed consent was obtained from all volunteers and the study was approved by the ethical boards of Bonn University. Donors were in average 38.8 (SD 9.6) years old and included 3 male and 9 female.

Primary human dermal fibroblasts were kindly provided be the Clinical Pharmacology Department of the University Medicine Göttingen (Dr. med. Markus Schirmer). Fibroblasts were isolated from healthy tissue removed by dermal excisions from patients at the clinic.

#### Cell culture

Keratinocytes were cultured in EpiLife medium with 1% human keratinocyte growth supplements (Gibco™, Waltham, MA, USA), 1% pen/strep and 0.1% amphotericin B, medium was changed every 2–3 days and cells were passaged when reaching a confluence of 75%. Fibroblasts were cultured in basal fibroblast growth medium 2 with supplement mix fibroblast growth medium 2 (2% FCS, 5 μg/ml Insulin, 0.001 μg/ml fibroblast growth factor (FGF)) (PromoCell, Heidelberg, Germany) and 1% pen/strep. The medium was changed every 2–3 days and cells were passaged when reaching a confluence of 75% every 5–7 days. Before miRNA profiling keratinocytes were incubated with 5μM erlotinib (SantaCruz Biotechnology, Dallas, TX, USA) or 0.05% DMSO for two hours and then stimulated with 4 nM EGF (PeproTech, Rocky Hill, NJ, USA) for 5 min. Fibroblasts were incubated with 5 μM erlotinib or 0.05% DMSO for 24 hours and then stimulated with 4nM EGF for 5 min.

### RNA isolation and miRNA profiling with Next Generation Sequencing

Total RNA from keratinocytes and fibroblasts was extracted using Trizol/ Chloroform. RNA was precipitated from the aqueous phase with isopropanol, resuspended in RNase-free water and stored at –80°C.

Library preparation for miRNA profiling was performed with the NEB Next Multiplex Small RNA Library Prep Set for Illumina (New England Biolabs, Ipswich, MA, USA) following the manufacturer’s protocol. Resulting cDNA library was purified with QiaQuick PCR purification kit (Qiagen, Hilden, Germany). A size selection of the generated cDNA was done with gel electrophoresis. 60 μl cDNA probes were mixed with 12 μl loading dye and 15 μl were pipetted into 5 pockets of an 10% TBE-Gel (Invitrogen by Thermo Fisher, Carlsbad, CA, USA) and separated for 1 h 20 min at 150 V in TBE buffer. Ethidium bromide was used for the visualization of the cDNA bands and the one at ~ 140 bp was cut out. Gel extraction of the cDNA was done following the manufactures protocol of the NEB Next Multiplex Small RNA Library Prep Set for Illumina. For the quantification and qualification of the size-selected cDNA the Agilent high sensitivity DNA Kit (Agilent, Santa Clara, CA, USA)) was used and analyzed with the Agilent Bioanalyzer 2100. MiRNA sequencing was performed with the MiSeq from Illumina using the MiSeq Reagent Kit v3 (Illumina, San Diego, CA, USA) following the manufactures protocol. A final concentration 15 pMol of cDNA was used.

### Analysis of sequencing data

Sequencing data was saved as fastq files which was then used for adapter trimming with the Cutadapt 1.9 software [53]. Alignment of the trimmed sequencing data to the human genome (build37) was performed using bowtie1.0 [54]. The same software was used for the aligment to published mature and precursor (hairpin) miRNAs collected in the miRBase data base (release 21). Read counts were normalised and miRNA expression was compared between keratinocytes/fibroblasts more reactive to erlotinib against less reactive cell samples. For further research miRNAs, which were significantly differentially expressed with a fold change ≥ 1.5 or a fold change ≤ 0.66 and a *p* value of 0.05 for keratinocytes or a *p* value of 0.01 for fibroblasts, were chosen. A cut of at a *p*-value of 0.01 was choosen for fibroblasts, because of the high amount of segnificant miRNAs at a cut of at a *p*-value of 0.05. For a better comparison of the three different treatments, Venn diagrams of differentially expressed miRNAS were generated. Data was uploaded on to the GEO database under the accession number GSE159602.

### Literature search to choose miRNAs for further experiments

Fifty-four miRNAs were significantly differentially expressed exclusively after erlotinib incubation. A literature search was used to choose the most promising miRNAs for further experiments in serum samples from EGFRI treated patients. For the search the PubMed database was used. Search terms consisted of the name of one of the 54 miRNAs together with “cancer”, “EGFR inhibitors” or “erlotinib”. We did not discriminate between miRNA found in blood samples or in tumor cells/biopsies and we included all EGFR inhibitor therapies.

### Analyzing miRNAs in patients of the dermatoxgen study

#### Study design

The Dermatoxgen study is a prospective, multicentric study with the aim to investigate pharmacogenetics factors of EGFR inhibitor induced skin toxicity. Patients with histologically confirmed solid tumors (pancreatic, colon, head and neck or non-small-cell lung cancer) who received an EGFR inhibitor (erlotinib, gefitinib, cetuximab or panitumumab) therapy for the first time were included in the Dermatoxgen study. Patient characteristics are shown in Tables 1 and 2. The study was approved by the ethical boards of Ulm University and the Ludwig-Maximilians-University of Munich and patients gave their written informed consent to participate. EGFRI admission was carried out according to approved indications, as previously described [22]. The appearance and severity of the skin rash were documented once a week after treatment start for 4 weeks. The severity of the skin rash was graded following the Toxicity Criteria for Adverse Events of the American National Cancer Institute (NCICTCAE version 3.0, 2006). Blood, serum and plasma samples were collected after 4 weeks of treatment. The skin rash was only treated reactively if it was necessary during the treatment. As treatment topical corticosteroids, topical antibiotics, oral antibiotics and antihistamines were used depending on the grade of skin rash. A preemptive treatment was excluded to avoid any bias which may arise from the suppressing effect of the preemptive treatment. Patients were followed-up for 12 months, with visits after 6 months and 12 months. The survival status at 360 days after initiation of EGFRI treatment was used for Kaplan-Meier analyses. Serum samples from 254 patients were used for the miRNA analysis.

### Collection of serum samples and miRNA extraction

Serum samples were collected during week 4 of the EGFRI treatment right before the admission of the next scheduled dose. About 7.5 ml blood were collected from each patient using blood-sampling tubes (Serum S-Monovette^®^, 7.5 ml, Sarstedt). After a rest of 20 min at room temperature blood samples were centrifuged at 2500× g for 10 min, aliquoted and stored immediately at –80°C.

miRNA was isolated with the miRNeasy serum/plasma kit (Qiagen, Hilden, Germany). Following the manufactures protocol 100 μl serum were used. 350 μl of the upper aquarious phase mixed with 525 μl 100% ethanol were pipetted onto the miRNeasy spin column. After several washing steps, RNA was eluted with 14 μl RNase-free water from the spin column.

### RT-qPCR

Isolated miRNA was transcribed into cDNA using the miScript RT kit (Qiagen, Hilden, Germany). 5 μl miRNA sample were processed following the manufacturer’s protocol using the HiFlex Buffer. After reverse transcription the samples were diluted in 80μl RNase-free water. qPCR was performed using the miScript primer assays (miR-21, miR-31, miR-520e, miR-17, miR-106b) from Qiagen (Qiagen, Hilden, Germany). A master mix was prepared consisting of miScript Universal primer, miScript Primer assay, SyberGreen kit and RNase-free water (Qiagen, Hilden, Germany) following the manufacturer’s protocol. Samples were analyzed as triplicates in a 364 well plate with the Light Cycler 480 from Roche (Roche, Basel, Switzerland). As reference, miRNA-93 (Qiagen, Hilden, Germany) was used.

## SUPPLEMENTARY MATERIALS



## Abbreviations

EGFRI: Epidermal growth factor receptor inhibitor
miRNA: micro RNA
NSLC: Non-small-lung-cancer
Head-and-neck-ca: head-and-neck-cancer
colon-ca: colon-rectal-cancer
pancreatic-ca: panctras-cancer
EGFR: epidermal growth factor receptor
ERFG: epiregulin
AREG: amphiregulin
SNP: Single Nucleotide Polymorphism
HGF: hepatocyte growth factor
PIK3CA: phosphatidylinositol-3-kinase
PHLPP1: PH domain and leucine-rich repeat protein phosphatase 1
EGFR-mAbs: epidermal growth factor receptor monoclonal antibodies
TKI: tyrosine kinase inhibitor
N: number of patients
NA: not known
dCP: delta crossing point
OS: overall survival
PTEN: phosphatase and tensin homolog
DFS: disease-free survival
TGFBR2: transforming growth factor-β receptor 2
TGF-β: transforming growth factor-β
TNF-α: tumor necrosis factor-α
