# Supplementary material for: Association between miRNA signatures in serum samples from epidermal growth factor inhibitor treated patients and skin toxicity
Source: Oncotarget. 2021 May 11;12(10):982–95. doi: 10.18632/oncotarget.27953 (PMC8121613; doi:10.18632/oncotarget.27953)
Supplement: Supplementary file 1 [file oncotarget-12-982-s001.pdf]

Association between miRNA signatures in serum samples from epidermal growth factor inhibitor treated patients and skin toxicity

SUPPLEMENTARY MATERIALS

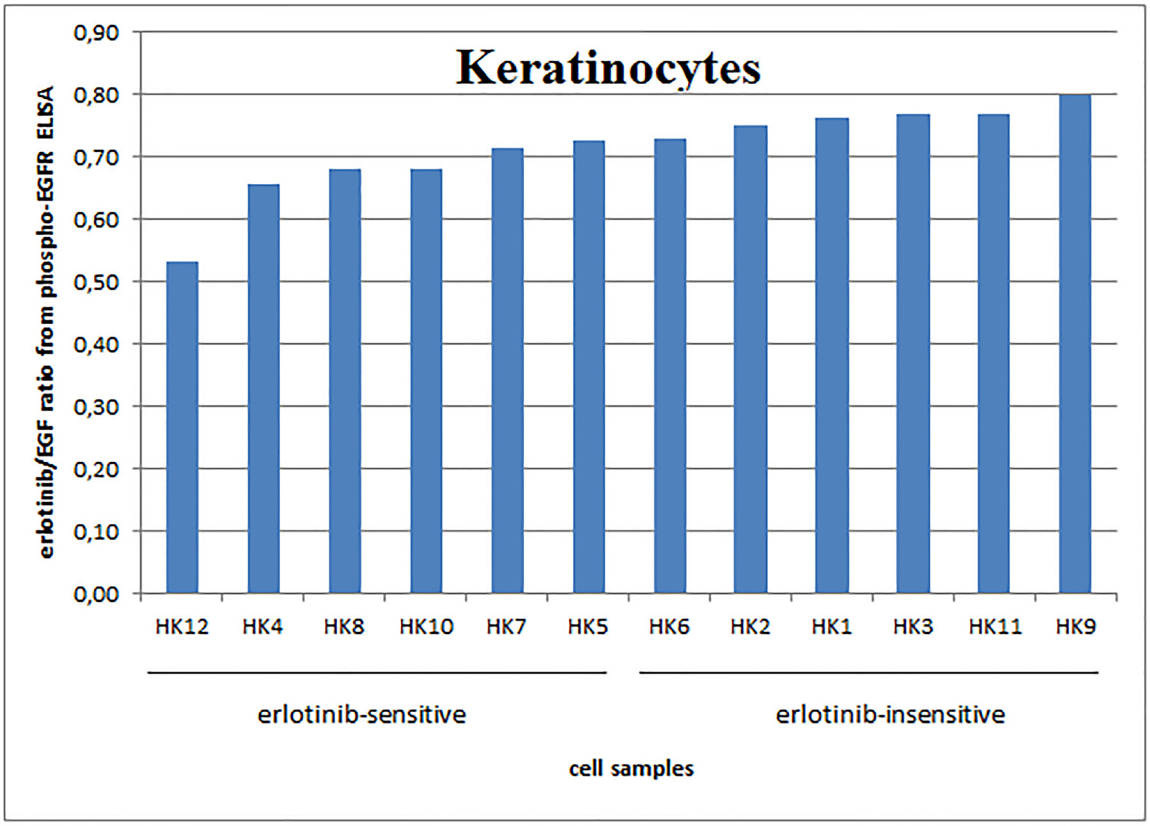

Supplementary Figure 1: Classification of primary human keratinocytes into cell lines reactive to erlotinib and cell lines less reactive to erlotinib. Total epidermal growth factor receptor (EGFR) amount and phosphorylated EGFR were assessed using a cell based human phosphor-EGF R (Y1068) immunoassay from R&D System.

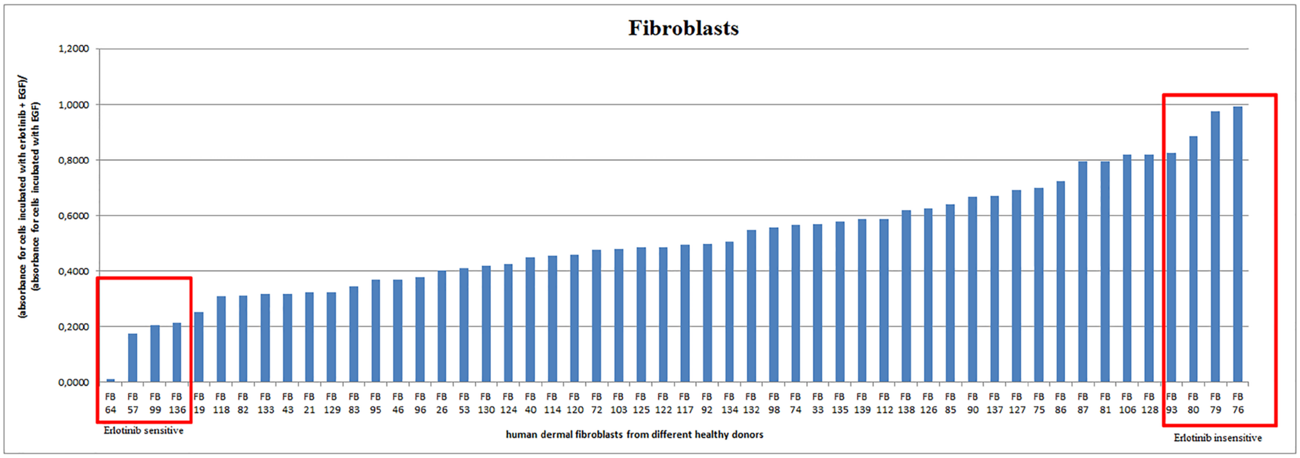

Supplementary Figure 2: Classification of primary human fibroblasts into cell lines reactive to erlotinib and cell lines less reactive to erlotinib. Total extracellular signal-regulated kinase 1/2 (ERK1/2) amount and the amount of phosphorylated ERK1/2 were assessed using a cell based human phosphor-EKR1/2 immunoassay from R&D System.

Supplementary Table 1: Table regulated miRNAs differentially expressed under at least one condition in Sensitive versus insensitive keratinocyte cells (≥ 1.50 (up-regulated) or ≤ 0.66 (down-regulated) $p < 0.05$)

	**erlotinib-sensitive vs. Insensitive incubation: none**		**erlotinib-sensitive vs. Insensitive incubation: EGF**		**erlotinib-sensitive vs. Insensitive incubation: erlotinib + EGF**	
**precursor miRNA (hairpin)**	fold change	*p*-value	fold change	*p*-value	fold change	*p*-value
hsa-mir-146a	0,64	0,0324	0,62	0,0440	0,55	0,0207
hsa-mir-944	1,11	0,4501	1,57	0,0103	1,67	0,0020
hsa-mir-203b	0,83	0,3688	0,62	0,0253	0,56	0,0129
hsa-mir-203a	0,83	0,3310	0,64	0,0266	0,60	0,0336
hsa-mir-3911	0,63	0,0044	0,89	0,5339	0,54	0,0158
hsa-mir-520e	0,81	0,3243	0,99	NA	0,18	0,0000
hsa-mir-138-2	0,72	0,1418	0,78	0,2511	0,40	0,0002
hsa-mir-585	1,10	0,6802	1,49	0,1046	2,15	0,0047
hsa-mir-1263	1,03	0,8875	0,73	0,1959	0,48	0,0082
hsa-mir-31	0,77	0,0423	0,79	0,0350	0,65	0,0096
hsa-mir-615	1,16	0,2588	1,10	0,5213	1,76	0,0110
hsa-mir-5690	1,09	0,5672	0,77	0,1899	0,55	0,0153
hsa-mir-3615	0,90	0,4332	1,02	0,9085	0,64	0,0156
hsa-mir-942	0,84	0,4482	1,39	0,1468	1,79	0,0168
hsa-mir-137	0,89	0,5282	1,01	0,9709	0,52	0,0174
hsa-mir-622	1,01	0,9356	0,80	0,1788	0,62	0,0236
hsa-mir-875	0,88	0,5562	0,95	0,8331	0,55	0,0268
hsa-mir-5195	0,76	0,2137	0,79	0,3352	0,55	0,0311
hsa-mir-675	1,29	0,1486	1,00	0,9899	0,60	0,0335
hsa-mir-609	0,92	0,4500	0,88	0,5610	0,60	0,0353
hsa-mir-711	0,99	0,9825	1,21	0,4496	0,61	0,0373
hsa-mir-6780b	1,03	0,7094	1,04	0,7154	0,63	0,0386
hsa-mir-6510	0,65	0,0594	0,68	0,1152	0,61	0,0493
hsa-mir-197	0,81	0,3128	1,06	0,7018	1,53	0,0500
hsa-mir-1181	1,02	0,9310	0,53	0,0040	0,80	0,3857
hsa-mir-4284	0,90	0,6430	0,54	0,0068	0,85	0,5583
hsa-mir-4461	0,80	0,3321	1,94	0,0071	0,90	0,6949
hsa-mir-9-3	0,99	0,9758	0,55	0,0144	0,93	0,7823
hsa-mir-8082	0,82	0,3888	0,57	0,0150	0,79	0,4035
hsa-mir-5000	0,87	0,5221	0,62	0,0186	0,83	0,4434
hsa-mir-877	0,83	0,4201	0,60	0,0310	0,93	0,7686
hsa-mir-3138	0,73	0,1167	0,62	0,0354	1,34	0,2883
hsa-mir-3973	0,98	0,9108	0,64	0,0430	1,25	0,3576
hsa-mir-6797	1,31	0,2248	0,63	0,0484	1,25	0,3811
hsa-mir-548ao	0,78	0,2453	1,57	0,0486	0,81	0,4391
hsa-mir-1246	1,01	0,9644	0,65	0,0491	0,84	0,4764
hsa-mir-1273c	0,66	0,0033	0,92	0,6016	0,81	0,3759
hsa-mir-95	1,67	0,0116	1,12	0,6452	1,15	0,5975

hsa-mir-611	0,65	0,0162	0,86	0,4288	0,99	0,9688
hsa-mir-5189	0,62	0,0191	0,82	0,3427	0,93	0,7764
hsa-mir-4451	0,62	0,0385	0,89	0,6300	1,24	0,4298
hsa-mir-485	0,62	0,0420	0,78	0,2954	0,88	0,6406
hsa-mir-4695	0,65	0,0461	0,74	0,1438	0,96	0,8778
hsa-mir-1973	0,64	0,0492	0,63	0,0568	0,79	0,3881

	**erlotinib-sensitive vs. Insensitive incubation: none**		**erlotinib-sensitive vs. Insensitive incubation: EGF**		**erlotinib-sensitive vs. Insensitive incubation: erlotinib + EGF**	
**mature miRNA**	fold change	*p*-value	fold change	*p*-value	fold change	*p*-value
hsa-miR-146a-5p	0,71	0,0258	0,57	0,0055	0,50	0,0040
hsa-miR-203a	0,83	0,3051	0,65	0,0254	0,61	0,0274
hsa-miR-520e	NA	NA	1,00	NA	0,38	0,0000
hsa-miR-138-5p	0,85	0,4073	0,90	0,6055	0,44	0,0005
hsa-miR-221-3p	0,91	0,4243	0,78	0,0497	0,66	0,0023
hsa-miR-138-2-3p	0,91	0,4976	0,82	0,2897	0,50	0,0047
hsa-miR-944	1,12	0,5161	1,49	0,0336	1,62	0,0054
hsa-miR-16-2-3p	1,33	0,0375	1,09	0,6038	1,76	0,0067
hsa-let-7e-3p	1,11	0,5806	0,92	0,7119	0,54	0,0127
hsa-miR-615-3p	1,16	0,2454	1,09	0,5805	1,68	0,0155
hsa-miR-210-3p	0,74	0,1254	0,81	0,2929	0,56	0,0175
hsa-miR-197-3p	0,85	0,3790	0,99	0,9521	1,60	0,0282
hsa-miR-7641	0,97	0,8657	0,81	0,3294	0,59	0,0314
hsa-miR-1282	1,04	0,8424	0,91	0,6629	1,56	0,0339
hsa-miR-1299	0,85	0,1580	0,85	0,2354	0,64	0,0414
hsa-miR-199a-3p	0,76	0,0852	0,61	0,0198	0,74	0,2122
hsa-miR-328-3p	0,83	0,3432	0,63	0,0348	0,81	0,3976
hsa-miR-744-3p	1,03	0,8511	1,55	0,0407	0,93	0,7555
hsa-miR-31-3p	0,59	0,0069	1,00	0,9889	0,90	0,6616
hsa-miR-4451	0,64	0,0123	0,97	0,8972	1,06	0,8196
hsa-miR-224-5p	1,53	0,0171	1,01	0,9378	1,12	0,5788

Supplementary Table 2: Table regulated miRNAs differentially expressed under at least one condition in Sensitive versus insensitive fibroblast cells (≥ 1.50 (up-regulated) or ≤ 0.66 (down-regulated) $p < 0.01$)

	erlotinib-sensitive vs. Insensitive incubation: none		erlotinib-sensitive vs. Insensitive incubation: EGF		erlotinib-sensitive vs. Insensitive incubation: erlotinib + EGF	
**precursor miRNA (hairpin)**	fold change	*p*-value	fold change	*p*-value	fold change	*p*-value
hsa-mir-101-1	1,7146	6,54E-03	1,7853	8,74E-03	1,6953	8,55E-03
hsa-mir-101-2	1,7984	3,60E-03	1,7853	8,74E-03	1,6994	8,24E-03
hsa-mir-103b-2	1,4733	6,90E-02	1,5637	2,65E-02	1,7633	8,63E-03
hsa-mir-106b	1,6011	1,81E-02	1,6811	7,98E-03	2,0366	2,56E-04
hsa-mir-107	1,6324	2,35E-02	1,8290	4,06E-03	1,7032	1,44E-02
hsa-mir-1-1	0,5216	6,02E-02	0,4371	2,52E-02	0,3451	4,05E-03
hsa-mir-1-2	0,6006	1,16E-01	0,6049	1,66E-01	0,3417	3,26E-03
hsa-mir-127	2,4273	8,93E-04	2,3501	5,51E-03	2,7086	3,39E-04
hsa-mir-1273a	0,6590	5,16E-02	0,4718	8,85E-04	0,7073	1,43E-01
hsa-mir-1273g	0,7296	1,60E-01	0,4810	8,97E-04	0,6859	1,15E-01
hsa-mir-134	1,6566	5,99E-02	1,9867	1,82E-02	2,3883	1,94E-03
hsa-mir-136	1,9258	1,02E-02	1,8743	4,29E-02	2,3141	3,50E-03
hsa-mir-146b	1,8397	2,38E-02	1,9176	2,47E-02	2,0270	6,56E-03
hsa-mir-148a	2,0042	4,29E-02	2,2684	6,72E-03	2,3654	2,46E-03
hsa-mir-148b	1,7997	2,70E-03	1,6924	2,26E-03	1,5005	4,11E-02
hsa-mir-155	1,7462	2,17E-02	1,9307	6,21E-03	2,0144	1,94E-03
hsa-mir-17	1,7806	7,49E-03	1,8057	2,05E-02	1,8541	1,08E-02
hsa-mir-199b	2,0187	3,79E-04	2,0153	7,66E-04	2,0322	5,23E-04
hsa-mir-20a	2,3367	7,68E-05	1,9294	7,42E-03	2,0163	2,37E-03
hsa-mir-25	1,4557	5,15E-02	1,6199	1,15E-02	1,6689	8,54E-03
hsa-mir-299	1,8224	2,72E-02	2,1532	1,41E-02	2,6276	5,12E-04
hsa-mir-30b	1,9416	4,90E-03	2,2175	1,34E-04	1,9906	1,92E-03
hsa-mir-3120	1,3885	1,38E-01	1,7755	9,18E-03	1,6782	2,28E-02
hsa-mir-323a	2,1035	4,32E-03	1,9785	2,77E-02	2,1822	4,58E-03
hsa-mir-342	1,9556	1,10E-02	2,3452	1,94E-03	1,6309	5,86E-02
hsa-mir-34a	1,5883	2,61E-02	1,7811	6,31E-03	1,7890	1,17E-02
hsa-mir-374b	1,6884	3,56E-02	1,6870	9,22E-03	1,3650	1,24E-01
hsa-mir-379	2,1823	4,80E-03	2,2720	6,85E-03	2,5382	5,40E-04
hsa-mir-382	2,4553	6,35E-04	2,4068	1,19E-03	2,3536	3,82E-03
hsa-mir-409	1,2938	4,54E-01	1,5128	1,90E-01	2,1711	8,87E-03
hsa-mir-411	2,0907	3,87E-03	1,6895	8,45E-02	1,6604	9,02E-02
hsa-mir-4508	0,5882	8,50E-02	0,4088	8,41E-03	0,7477	4,08E-01
hsa-mir-450b	2,0298	1,61E-03	1,9546	2,00E-03	1,7143	1,39E-02
hsa-mir-4510	0,6050	3,86E-02	0,5629	4,72E-03	0,4986	1,61E-03
hsa-mir-452	1,7670	3,27E-02	2,4547	1,98E-03	2,4260	9,78E-04
hsa-mir-494	2,7333	9,21E-05	2,4921	2,96E-03	2,6972	4,77E-04
hsa-mir-520e	0,3208	8,31E-04	0,4511	3,00E-02	0,3969	1,28E-02
hsa-mir-550a-3	0,5681	4,91E-02	0,5654	7,26E-02	0,4003	4,64E-03

hsa-mir-585	1,9586	1,25E-02	2,1195	5,89E-03	2,0655	8,01E-03
hsa-mir-615	0,6869	5,01E-02	0,5764	6,17E-03	0,6045	1,84E-02
hsa-mir-654	2,0690	3,88E-03	1,7210	5,26E-02	2,0419	1,02E-02
hsa-mir-7112-2	0,7921	4,64E-01	1,1570	4,35E-01	1,7932	6,72E-03
hsa-mir-7158	1,7918	1,88E-02	1,8522	2,36E-02	2,4108	8,32E-04
hsa-mir-889	2,3618	1,72E-03	1,9932	1,76E-02	2,3003	1,89E-03

	**erlotinib-sensitive vs. Insensitive incubation: none**		**erlotinib-sensitive vs. Insensitive incubation: EGF**		**erlotinib-sensitive vs. Insensitive incubation: erlotinib + EGF**	
**mature miRNA**	fold change	*p*-value	fold change	*p*-value	fold change	*p*-value
hsa-let-7d-3p	0,7102	2,16E-01	0,5184	1,02E-03	0,7829	1,37E-01
hsa-miR-106b-3p	1,3139	9,44E-02	1,3310	4,80E-02	1,6990	7,29E-04
hsa-miR-125b-1-3p	0,6516	3,27E-02	0,6542	1,12E-03	0,7267	1,55E-02
hsa-miR-1273d	0,7335	4,56E-01	0,2853	3,29E-03	0,8231	6,26E-01
hsa-miR-1273g-3p	0,5914	1,16E-01	0,3630	2,17E-03	0,6850	2,47E-01
hsa-miR-127-5p	2,1115	2,67E-02	2,8260	2,30E-03	2,2678	8,79E-03
hsa-miR-1285-5p	0,7328	4,38E-01	0,2778	1,14E-03	0,4370	8,05E-03
hsa-miR-1290	0,5930	1,19E-01	0,4699	2,36E-02	0,4531	2,17E-03
hsa-miR-1303	0,5535	2,52E-02	0,4850	8,58E-03	0,6247	4,58E-02
hsa-miR-138-5p	0,4441	8,50E-04	0,5585	2,72E-03	0,5569	3,68E-03
hsa-miR-140-5p	0,6952	6,24E-02	0,6653	3,27E-03	0,5546	6,94E-06
hsa-miR-145-3p	0,3568	1,91E-03	0,3834	1,15E-02	0,4342	9,35E-03
hsa-miR-148a-3p	2,4485	7,43E-03	1,9506	5,37E-02	1,8947	5,10E-02
hsa-miR-149-3p	0,6838	3,44E-01	0,2749	6,69E-03	0,8065	5,89E-01
hsa-miR-17-5p	1,6183	1,68E-02	1,6490	1,34E-02	1,8342	5,65E-03
hsa-miR-1827	0,6129	1,86E-01	0,3533	1,71E-03	0,3823	2,88E-03
hsa-miR-199b-5p	2,7918	1,35E-03	2,6238	4,86E-03	2,3124	1,32E-02
hsa-miR-20a-5p	2,0512	1,13E-03	1,7428	3,69E-03	1,8640	2,70E-03
hsa-miR-22-3p	0,5338	2,75E-03	0,5946	2,38E-08	0,5465	5,67E-07
hsa-miR-22-5p	0,5061	1,75E-04	0,5798	5,18E-03	0,5603	7,68E-04
hsa-miR-27b-5p	0,4562	6,70E-03	0,5060	1,89E-02	0,7599	3,53E-01
hsa-miR-299-3p	1,7657	4,86E-02	2,3847	3,43E-03	2,8374	2,03E-05
hsa-miR-300	1,2825	5,53E-01	3,8119	4,53E-03	1,6666	2,04E-01
hsa-miR-30b-5p	1,8645	6,53E-03	1,9837	3,84E-03	1,7778	7,76E-03
hsa-miR-3135b	0,5089	1,98E-02	0,4636	5,36E-03	0,6078	6,72E-02
hsa-miR-361-3p	1,6441	7,59E-03	1,3161	1,33E-01	1,7261	9,52E-04
hsa-miR-3976	0,9944	9,85E-01	0,5879	9,19E-02	0,5263	4,47E-03
hsa-miR-409-5p	2,2387	2,56E-02	1,9141	7,98E-02	2,4874	8,84E-03
hsa-miR-4485	0,3485	4,06E-03	0,3827	2,28E-02	0,5413	1,10E-01
hsa-miR-450b-5p	1,8012	2,43E-03	1,6657	2,31E-04	1,5094	1,58E-02
hsa-miR-4510	0,5287	1,82E-02	0,4535	1,00E-04	0,4160	1,35E-07
hsa-miR-452-5p	1,8438	6,19E-02	4,5281	2,67E-05	1,7737	6,54E-02
hsa-miR-4648	0,6744	3,52E-01	0,2610	4,97E-03	1,1646	7,01E-01
hsa-miR-4765	0,9848	9,66E-01	0,7366	3,96E-01	0,4281	9,28E-03
hsa-miR-490-3p	0,4608	5,28E-02	0,2605	4,76E-03	0,5468	1,37E-01

hsa-miR-504-5p	3,6075	2,23E-04	1,6152	1,92E-01	1,7851	8,57E-02
hsa-miR-548h-5p	1,1158	7,96E-01	3,2007	7,33E-03	1,3682	3,98E-01
hsa-miR-5585-3p	0,8276	5,82E-01	0,4134	8,98E-03	0,6542	1,36E-01
hsa-miR-5684	0,4730	2,74E-02	0,3421	1,56E-03	0,6211	1,36E-01
hsa-miR-615-3p	0,5987	4,07E-02	0,4624	2,79E-03	0,5439	1,43E-02
hsa-miR-628-5p	1,3757	4,53E-01	1,1009	8,29E-01	0,3535	8,03E-03
hsa-miR-651-5p	4,1021	1,36E-03	1,4373	4,13E-01	1,3346	4,65E-01
hsa-miR-653-3p	0,7501	4,37E-01	0,3504	7,71E-03	0,4718	1,15E-02
hsa-miR-654-5p	2,5440	1,94E-03	2,1584	2,30E-02	1,8405	6,52E-02
hsa-miR-655-3p	2,8461	2,40E-03	2,3518	1,15E-02	1,7716	8,90E-02
hsa-miR-6756-5p	0,2458	4,60E-04	0,3562	2,31E-02	0,5013	5,43E-02
hsa-miR-758-3p	1,9017	1,40E-02	1,7206	9,88E-02	1,9454	5,70E-03
hsa-miR-7641	0,4237	9,31E-03	0,5404	9,77E-02	0,5998	1,00E-01
